# A Comparison of Capacitive Soil Moisture Sensors in Different Substrates for Use in Irrigation Systems

**DOI:** 10.3390/s25051461

**Published:** 2025-02-27

**Authors:** Mehmet Akif Gümüser, Alexander Pichlhöfer, Azra Korjenic

**Affiliations:** Research Unit of Ecological Building Technologies, Institute of Material Technology, Building Physics and Building Ecology, Faculty of Civil and Environmental Engineering, Vienna University of Technology, 1040 Vienna, Austria; mehmet.guemueser@tuwien.ac.at (M.A.G.); azra.korjenic@tuwien.ac.at (A.K.)

**Keywords:** moisture sensor, intelligent irrigation, intelligent watering, greening system, capacitive soil moisture sensors

## Abstract

Smart irrigation systems play a crucial role in water management, particularly in urban greening applications aimed at mitigating urban heat islands and enhancing environmental sustainability. These systems rely on soil moisture sensors to optimize water usage, ensuring that irrigation is precisely tailored to plant needs. This study evaluates the performance of four commercially available capacitive soil moisture sensors—TEROS 10, SMT50, Scanntronik, and DFROBOT—across three different substrates under controlled laboratory conditions. A total of 380 measurements were conducted to assess sensor accuracy, reliability, and the influence of insertion technique on measurement variability. Results indicate that while all sensors adequately cover the moisture ranges critical for plant health, their accuracy varies significantly, highlighting the necessity of substrate-specific calibration. TEROS 10 exhibited the lowest relative deviation and highest measurement consistency, making it the most reliable among the tested sensors. DFROBOT, despite being the least expensive, performed comparably to SMT50 and Scanntronik in certain conditions. The findings provide valuable insights for selecting and calibrating soil moisture sensors in smart irrigation applications, ultimately contributing to improved water efficiency, plant vitality, and sustainable building-integrated greenery.

## 1. Introduction

**Figure 1:**
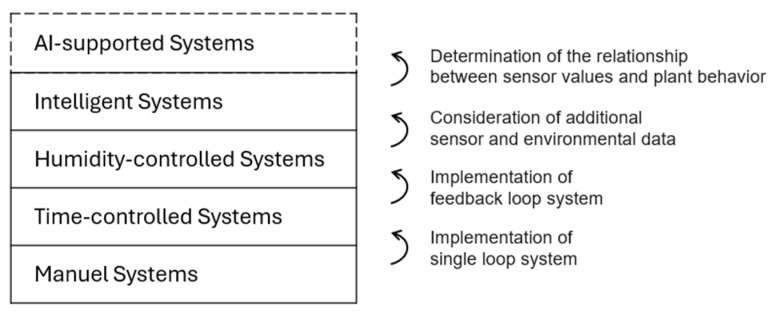
Five levels of the irrigation system, considering the control, regulation, and AI technology (own representation).

The longevity and functionality of interior and exterior greening of buildings can be improved using irrigation systems. A well-designed and intelligent irrigation system allows plants to thrive optimally, thereby maximizing their growth and vitality, which is essential not only for the aesthetics of the building envelope but also for the effect on building physics and microclimatic effects to provide benefits to the urban environment [1,2]. The purpose of the irrigation system is to ensure the most efficient use of water by applying the right amount of water at the right time and in the right spot [3]. We categorize irrigation systems in terms of control and regulation technology, as shown in Figure 1.

We consider Intelligent Systems and AI-supported Systems as smart irrigation systems, which are designed to determine the most suitable irrigation schedule for plants by processing data received from soil/substrate moisture sensors and optionally other climate-related and stress-related plant data, mainly with the goal of reducing water and energy consumption. For example, by implementing smart irrigation, 20% of water usage can be reduced by maintaining soil moisture from 0.13 to 0.22 m^3^/m^3^ (13 to 22% VWC) [4]. Since soil moisture serves as a solvent and carrier of minerals and organic matter, regulates soil temperature, and plays a vital role in chemical and physical processes, it has a direct influence on plant health and development [5]. Therefore soil moisture is more immediate compared to other environmental factors, suggesting a paramount importance over other environmental variables like air temperature [6].

**Figure 2:**
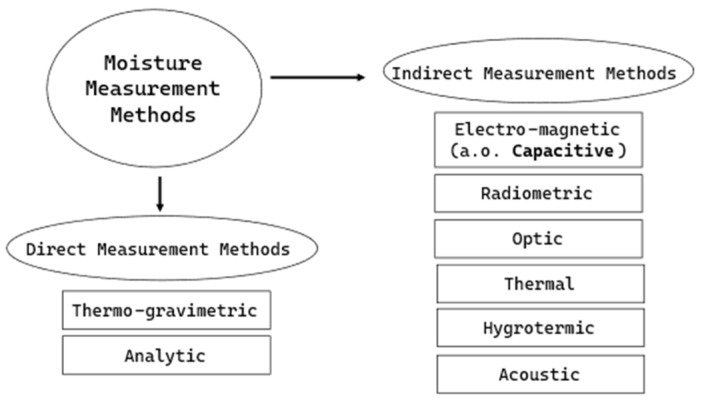
Classification of material moisture measurement methods according to (Ref. [9], S. 49).

There are many different methods and techniques for detecting moisture in material. All common measuring methods shown in Figure 2 can be used to measure soil moisture ([7], S. 205). Direct and indirect methods provide qualitative and/or quantitative results. A compilation of the principles of soil moisture measurement methods can be found in the following literature: [8].

In this study, only capacitive sensors were considered, which fall under the category of stationary, indirect, invasive, in situ proximal soil sensors, since sensors are not moved during measurement, they measure electro-magnetic properties, they have direct soil contact during measurement, and the measurements are made within the soil [10]. Generally, capacitive moisture sensors are preferred since they offer non-destructive measurement, real-time monitoring, cost-effectiveness, and low power consumption [11]. The development of capacitive moisture sensors dates to the early 1990s. A significant study from 1993 examined the properties of capacitive thin-film moisture sensors, laying the foundation for further research in this field [12]. Thin-film capacitive moisture sensors operate by measuring the change in capacitance of a capacitor and typically consist of two conductive electrodes separated by a dielectric material that reacts to changes in moisture by altering its relative permittivity. Thus, an increase in moisture is leading to an increase in capacitance [13]. However, these sensors have the main drawback that aging and contamination of the dielectric material can lead to a significant loss of accuracy. A more prevalent and resilient design for capacitive soil moisture sensors is making use of so-called fringe field capacitors [14], where an electric field, produced by electrodes, extends into the surrounding soil and the capacitance of the soil is directly measured via techniques like Frequency Domain Reflectometry (FDR) [15]. In FDR, an oscillating electrical signal is applied, and the resulting frequency response is analyzed to determine the soil’s dielectric constant, which correlates with the volumetric water content of the soil, allowing for moisture estimation through post-processing. Another similar method to measure the capacitance is Time Domain Reflectometry (TDR) [16], where the dielectric permittivity of the soil is determined by sending an electrical pulse along a transmission line (typically metal rods or waveguides embedded in the soil) and measuring the time it takes for the pulse to reflect back [17]. Understanding the diversity of these sensors and their designs and configurations, each tailored to specific applications and requirements can be crucial for selecting the appropriate type for a given use case. However, in general, capacitive sensors lack good performance in environments with voids or inhomogeneities due to lack of contact and are further influenced by temperature, soil composition, salinity, and the available measurement volume. Since greening systems generally use substrates with a homogeneous mixture, the use of capacitive sensors is preferred and practically suitable if enough soil- or substrate volume is provided. Further, not every (capacitive) sensor performs adequately in every type of substrate and sensors often require thorough calibration processes due to their reliance on soil characteristics [18]. These calibration processes often include the need to prepare soil- or substrate samples with different, known moisture contents and the repeated installation of each sensor.

When placed in the substrate, differences in tightness and insertion depth have a significant influence on the capacitive sensor’s measurement and output. Although it is easy to ensure a uniform and reproducible insertion depth, it is almost impossible to reproduce the tightness of the soil or substrate without using a special method that must be developed first, which means that the contact of the soil or substrate always has a decisive influence on the measurement. As a result, it is practically challenging to reproduce and verify calibration parameters and accuracies provided by manufacturers. Regarding the application in automatic irrigation systems, it can, therefore, be assumed that each individual sensor must be considered and evaluated individually, preferably after installation. In general, the suitability of these sensors and their application can, therefore, be questioned, which is leading to reoccurring discussions in the field of environmental engineering, where moisture sensors are applied but not necessarily well understood. Apart from this problem, the suitability of the sensors for the designated task still can be judged. For this purpose, the range of moisture in which plant-available water is present, the permanent wilting point, and the point at which the field capacity is reached should be considered. The permanent wilting point describes the condition when there is no water available to the plant and water uptake is restricted. It depends on plant variety but is usually defined as around 15 bars of matric potential. At this point, the soil still contains water; however, it is difficult or impossible for plants to extract moisture from the soil. At this limit, plants take irreversible damage if no additional water is supplied to the soil or substrate. The moisture content at the permanent wilting point varies with soil texture and soil composition. Fine-textured soils retain higher amounts of water (~26–32% VWC) than coarse-textured soil (10–15% VWC) at the permanent wilting point [19]. Field capacity is the VWC at which saturation is reached, and no additional water can be held against gravity. The quantity of water a plant requires is influenced by several factors. These include the plant’s species and morphology, environmental aspects such as sunlight exposure, ambient temperature, and air moisture levels, as well as the soil’s characteristics and the material and size of the container in which the plant is grown [20]. The results of this study [21] show that plants remain healthy when soil moisture is kept between 50 and 70% VWC. Another experiment analyzed the relationship between different soil moisture content and plant growth [22].

In this study, commercially available capacitive soil moisture sensors were examined under laboratory conditions, and the resulting values were compared. In the results, we empathize with the reproducibility of sensor values regarding the sensor insertion process and compare the resulting calibration equations to equations provided by the manufacturers. Finally, we judged the suitability of the sensors by comparing their performance with the suction tension curve for one type of substrate. This allows a statement to be made as to whether the moisture range covered by the respective sensor and the inaccuracies stemming from differences in insertion is suitable for correctly assessing the relevant moisture situation of plants in a substrate. This comparison can be helpful for developers and users of smart irrigation systems when choosing sensors for irrigation systems for the rooftop or vertical greening of the built environment.

## 2. Materials and Methods

### 2.1. Soil Moisture Sensors and Substrates Used in Measurements

**Figure 3:**
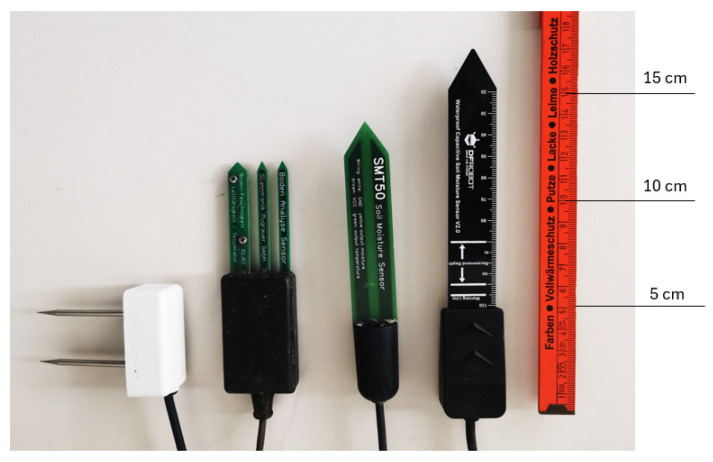
Soil moisture sensors used in measurements.

This study examined the performances of four different brands of capacitive sensors on the market in three different substrate mixtures. We selected both cheap and expensive sensors that are easily available in the market. The selected sensors are listed in Table 1 and shown in Figure 3.

**Figure 4:**
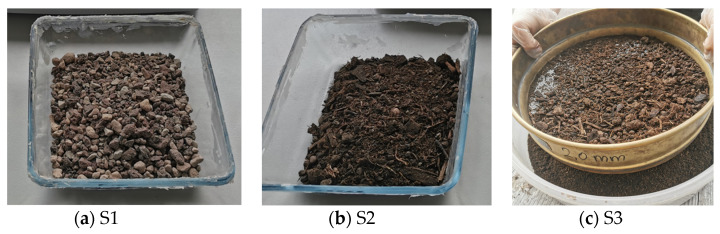
Substrates: Zeobon (**a**), Kranzinger (**b**), Kranzinger sieved through 2 mm (**c**).

Images of the soil moisture sensors used in the measurements are shown below:

The sensors were placed in three different substrates. The first substrate “Zeobon^®^ (Zeostrat 2/8)” includes lava, pumice, and zeolite (Zeobon GmbH, Dattenberg, Germany [23]) marked as S1 in the following chapters. The second substrate, “Kranzinger (trough and roof substrate)”, consists of white peat, quality compost, bark humus, expanded clay, wood fiber, foam lava, brick chippings, clay minerals, and mineral multi-nutrient fertilizer (Franz KRANZINGER GmbH, Straßwalchen, Austria [24]) and will be called S2 in the following chapters.As a third substrate, the Kranzinger substrate was prepared by sieving it through a sieve with a mesh size of 2 mm to minimize the voids in the substrate, caused by organic matter like bark and will be called S3 in the following. All three substrates are shown in Figure 4 below. The vegetation parameters for S1 and S2, provided in the manufacturer datasheets, are shown in Table 2.

### 2.2. Measuring Process

During measurements, the behavior and response to different moisture levels of four different sensors were evaluated in three different substrates, as shown above. Measurements require a drying oven, mass balance, calibration container, mixing container, and drying containers and were carried out in a lab environment with a constant temperature of 20–22 °C and humidity of 40–50% relative humidity.

**Figure 5:**
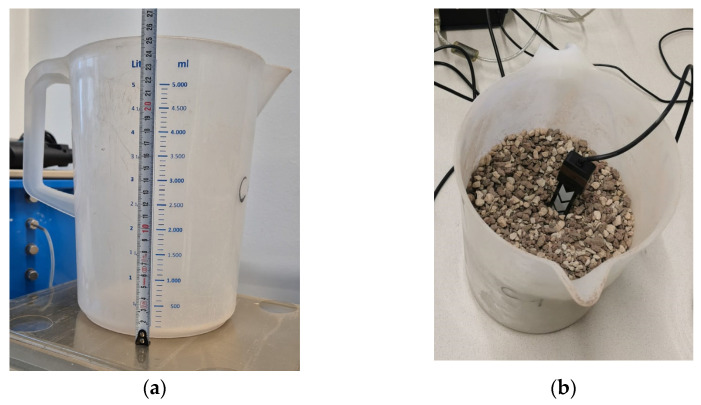
Calibration container (**a**) and sensor insertion (**b**).

The method for analyzing the sensors is based on a method provided by the manufacturer METER [25] and was adapted and examined as described in detail below. First, a container for the measurements had to be chosen according to manufacturer recommendations in relation to the measurement volume. Since we have only found recommendations provided for TEROS 10, which is 430 mL [26], we adapted the requirements for all other examined sensors and carried out all measurements in a 5000 mL beaker to eliminate possible influences caused by too low substrate volume (Figure 5a). The dimensions of the beaker were height = 25.5 cm, diameter (top) = 20 cm, diameter (bottom) = 18 cm (Figure 5). Each individual sensor was placed in the beaker one after the other. To do this, the beaker was first filled with substrate up to the 2500 mL mark; then, each sensor was inserted with the electrodes facing the bottom of the container and then filled with substrate up to the 5000 mL mark (Figure 5b). The installation depth was, therefore, as shown in Table 3. After each installation, the compression of the substrate was carried out by hand to ensure a constant substrate volume of 5000 mL ($V_{substrate})$.

This ensured a reproducible installation of the sensor and guaranteed a sufficient volume of substrate surrounding the sensors. The data were then recorded for each sensor for 1 min at intervals of 5 s with the help of data loggers and the mean value of this data was then used as the resulting sensor value. To add water evenly to the substrate and obtain individual increasing moisture levels, the sensor was removed after each measurement, the substrate was then transferred to a larger container, and the appropriate amount of water $m_{H_{2}O}$ was added as evenly as possible using a standard household spray bottle, while the substrate was mixed by hand. Each measurement series starts with the moisture level of the air-dried substrate. The air-dried substrate was prepared by letting each substrate dry for 5 days at 20–22 °C by putting 6 liters each into open containers. To determine the remaining water content, we took three samples of each air-dried substrate after mixing it thoroughly and dried the samples at 60 °C until constant mass was reached. This step was carried out for each substrate immediately before we started with the corresponding measurement series.

The increments ($m_{H_{2}O}$) in which water was added were determined by considering the approximate maximum saturation of each substrate. For S1, according to the specification given by the manufacturer, the maximum water capacity is 21% VWC [23]. For S2, the maximum water capacity was determined with the help of a pressure plate extractor in a project we conducted earlier (compared with [27]). Since we modified S2 to obtain S3, the maximum water capacity was unknown, and we chose 25% VWC as the upper limit. Therefore, water was added in steps of approximately 3% VWC for S1, S3 and 10% VWC for S2.

Once the dedicated amount of water had been added for each intermediate step, the substrate was mixed again thoroughly by hand and left to stand for approximately 5 min. The substrate was then transferred back into the 5000 mL beaker, again, first up to half and then up to the 5000 mL mark, and the sensor was reinstalled at the same time. For each sensor and moisture level, the sensor was installed and removed in the same way four times. For this, the substrate was also transferred to the mixing container and then refilled into the 5000 mL beaker in two steps (without adding water) while the sensor was inserted again. To determine the (initial) water content of the air-dried substrate, $\theta_{airdry}$ using Equation (3), the mass of each sample after drying in the oven $m_{substrate,\;oven\;dry}$ was determined, and $w_{H_{2}O,\;air\;dry}$ was calculated with the help of Equations (1) and (2). Since three samples were dried, an average of $w_{H_{2}O,airdry}$ was calculated. We also determined the mass of the 5000 mL air-dried substrate ${(m}_{substrate,\;air\;dry,5000})$ and the mass of the 5000 mL substrate for each moisture level ($m_{substrate,\;wet,5000}=m_{substrate,\;air\;dry,5000}+m_{H_{2}O,n})$, where n is each moisture level we examined. This was carried out to calculate $\theta_{n}$, the volumetric water content for each moisture level, using Equations (4) and (5).

(1) $$m_{H_{2}O,substrate,airdry}(kg)=m_{substrate,airdry}(kg)-m_{substrate,ovendry}(kg)$$

(2) $$w_{H_{2}O,airdry}(\%)=\frac{m_{H_{2}O,substrate,airdry}(kg)}{m_{substrate,airdry}(kg)}$$

(3) $$\theta_{airdry}(\%)=\frac{m_{substrate,airdry,5000}\times w_{H_{2}O,airdry}\times \rho_{H_{2}O}(998.2\frac{kg}{m^{3}})}{V_{substrate}(0.005m^{3})}$$

(4) $$m_{H_{2}O,n}=m_{substrate,\;wet,5000}-m_{substrate,\;air\;dry,5000}$$

(5) $$\theta_{n},\%=\frac{m_{H_{2}O}\times \rho_{H_{2}O}(998.2\frac{kg}{m^{3}})}{V_{substrate}}$$

## 3. Results

**Figure 6:**
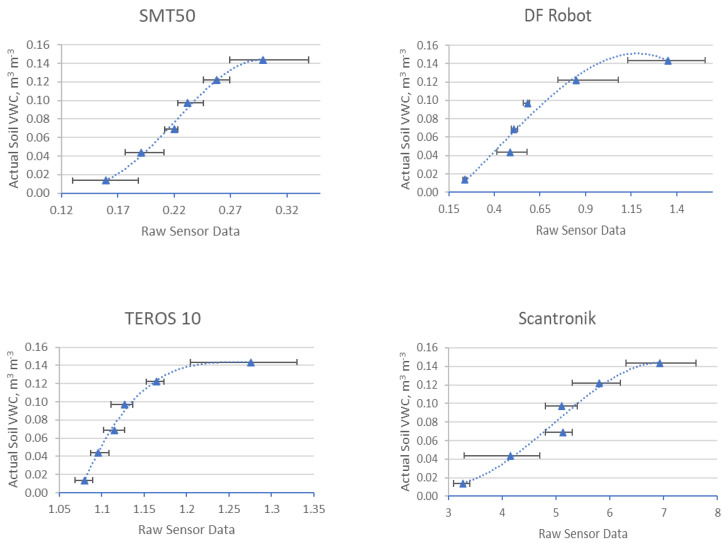
Comparison of four different sensors in S1.

**Figure 7:**
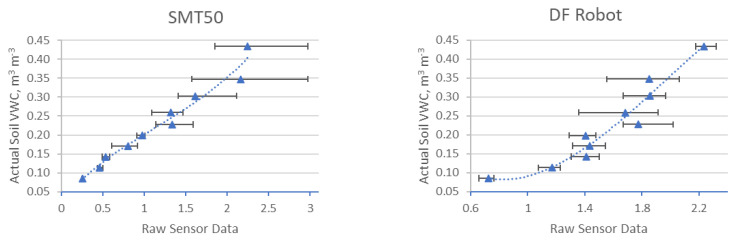

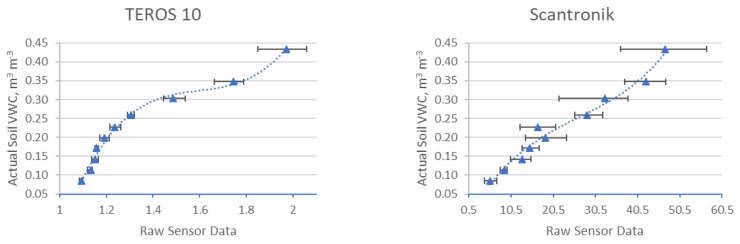
Comparison of four different sensors in substrate S2.

**Figure 8:**
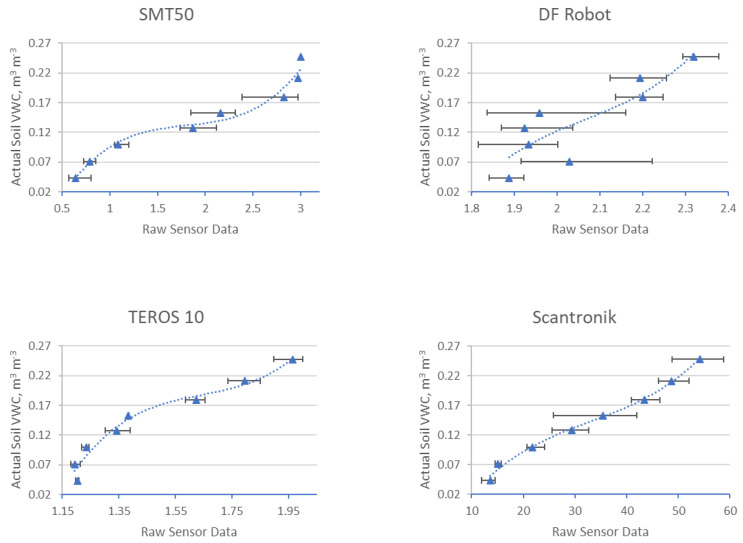
Comparison of four different sensors in S3.

In total, 380 measurements were made with four different sensors in three different substrates. Each data point on the graphs in Figure 6, Figure 7 and Figure 8 is derived from the average of four measurements. The lines show the range of minimum and maximum values for each average, and the corresponding data are shown. The sensor readings are shown in the horizontal axis and the VWC values ($\theta_{airdry},\;{\;\theta }_{n})$ of the substrates are shown in the vertical axis. The results of these sensor measurements and the fitting functions are shown below. First, measurements conducted on S1 are presented in Figure 6, followed by S2, Figure 7, and, finally, S3, Figure 8. After each graph, the calibration functions and the coefficient of determination R^2^ are compared in the tables.

### 3.1. Results of Four Different Sensors in S1

The results of these sensor measurements in S1 and the fitting functions are shown in Figure 6 below.

Table 4 shows the values of the individual measurements, the corresponding mean value (Ø), as well as the standard deviation (σ). Table 5 shows the coefficients and the coefficient of determination (R^2^) values for each sensor calibration function derived from the measurements conducted in substrate S1.

### 3.2. Results of Four Different Sensors in S2

The results of these sensor measurements in S2 and the fitting functions are shown in Figure 7 below.

Table 6 shows the values of the individual measurements, the corresponding mean value (Ø), as well as the standard deviation (σ). Table 7 shows the coefficients and the coefficient of determination (R^2^) values for each sensor calibration function derived from the measurements conducted in the S2.

### 3.3. Results of Four Different Sensors in S3

The results of these sensor measurements in S3 and the fitting functions are shown in Figure 8 below.

Table 8 shows the values of the individual measurements, the corresponding mean value (Ø), as well as the standard deviation (σ). Table 9 shows the coefficients and the coefficient of determination (R^2^) values for each sensor calibration function derived from the measurements conducted in S3.

To have an overview of the measurements conducted and judge which sensor has the insertion-dependent reproducibility of sensor values, we calculated the average ($x_{avg})$ of the relative deviations σ (%) shown in the tables above. These values are shown in Table 10, Table 11 and Table 12. As can be seen, TEROS 10 has the least average relative deviation $\left(x_{avg}\right)$ and the lowest range of relative deviation ($x_{\min}-x_{max})$ in all substrates, which leads to the conclusion that the sensor delivers reproducible values when similar sensor insertion technique is retained. Surprisingly, DFROBOT, which is the cheapest of the examined sensors, has the second lowest values for $x_{avg}$ whereby the differences to SMT50 and Scanntronik in S3 are not significant. SMT50 and Scanntronik show quite similar values for $x_{avg}$, with SMT50 performing slightly better in S2 and S3. It should be noted that, in most cases, the range of relative deviation ($x_{\min}-x_{max}$) is so large that the differences between sensors are not significant. If, for example, the σ of $x_{avg}$ in S1 for SMT50 is compared with DFROBOT, it becomes clear that no reliable statement can be made for these sensors in this substrate with the chosen installation technique. In S2, SMT50 shows a lower $x_{avg}$ but a higher σ thereof, in comparison to Scanntronik. Comparing the σ of $x_{avg}$ of SMT50 with DFROBOT, it becomes clear that SMT50 could perform as good as DFROBOT or DFROBOT could perform as bad as SMT50. In S3, all sensors besides TEROS10 performed very similarly, even when considering the σ of $x_{avg}$.

## 4. Discussion

**Figure 9:**
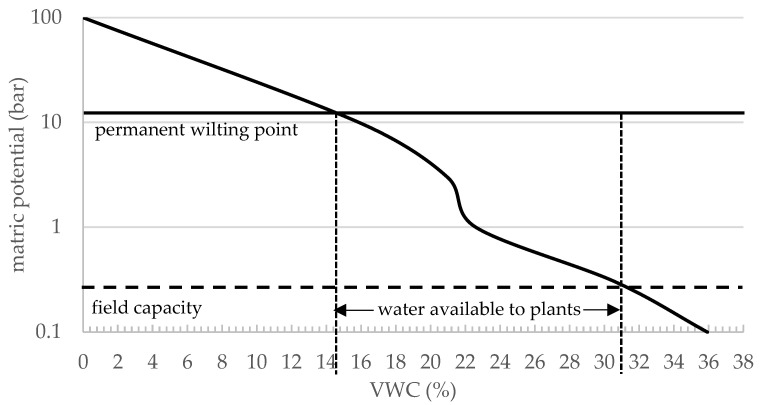
Suction tension curve (matric potential vs. volumetric water content) for S2 [27].

In the discussion, we consider that the main subject of this article is the application of capacitive sensors in irrigation systems. Additionally, to the comparison of each other, we compared the equations we derived from our experiments with the equations that are provided by the manufacturers. Since no equations are provided for DF Robot and Scanntronik sensors, only equations for TEROS 10 and SMT 50 were examined. In the following, each sensor is described and discussed in more detail. In a previous project, the suction tension curve for S2 was determined [27], as shown in Figure 9, which further allows us to discuss the performance of the sensors in this type of soil in more detail.

### 4.1. SMT 50

For SMT 50, a linear equation is provided within the manual, where U is the sensor output in volts (Equation (6)).

(6) $$\theta =VWC\left(\%\right)=\left(U\left(V\right)\times 50\right)÷3$$

**Figure 10:**
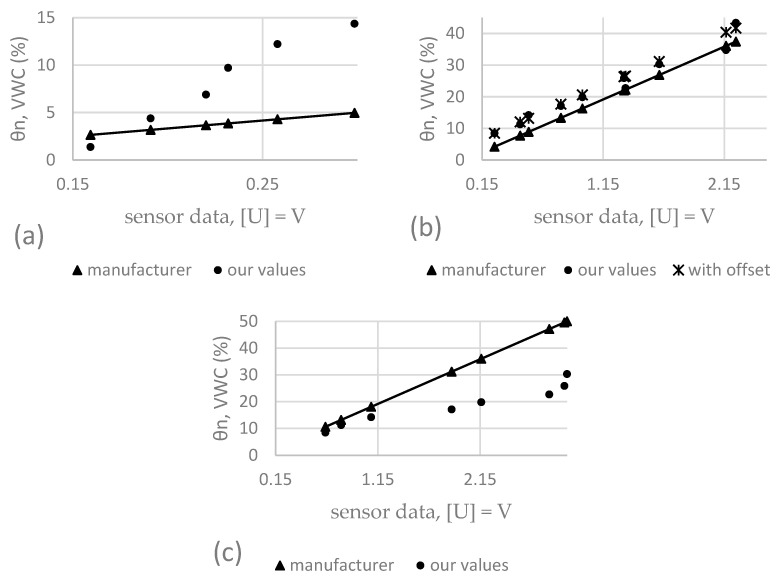
Measured and calculated VWC for SMT 50 in substrates S1 (**a**), S2 (**b**), and S3 (**c**).

Table 13 shows the calculated values for $\theta_{n}$, VWC (%) in substrate S2 in comparison to each other. Both datasets show a very comparable slope, and there seems to be an offset (systematic error) present. This error can be caused by the aforementioned fact that the measurements are highly dependent on the environment and sensor installation/insertion conditions, as well as the design of the experiment. To show this, we added an offset to the dataset derived from the manufacturer’s equation and plotted it in Figure 10b. To keep things simple, the offset was only derived from one single data point at which the substrate was air-dried, and no additional water had been added yet. When compared to the suction tension curve in Figure 9, both equations lead to the assumption that the range of available water is covered by the sensor and the deviations would not lead to serious misinterpretation of the VWC present, since the usage of the manufacturers equation would lead to an underestimation of the actual VWC at the wilting point. At the field capacity, however, this underestimation would lead to unnecessary additional watering, since the substrate would not be able to hold more water, and the surplus would go directly to the drain, washing out nutrients and fine the components of the substrate. When looking at the comparison for substrates S1 (Figure 10a) and S3 (Figure 10c), a completely different result was obtained. As can be seen, the difference between the calculated curves cannot be described by an offset that is added to the respective values. Instead, it is easy to see that the slope of the curves would have to be adjusted, and the manufacturer’s equation would lead to a significant underestimation of VWC in the case of S1 and an overestimation in the case of S3. The respective values are shown in Table 13 and the calculated average deviations are shown in Table 14 and underline the fact that the performance of the sensor varied depending on the substrate, with 4.8 ± 3.2 for S1, 3.6 ± 1.5 for S2 and 13.2 ± 8.8% VWC for S3.

### 4.2. TEROS 10

For TEROS 10, a cubic formula is provided within the manual, shown in Equation (7).

(7) $$VWC\left(\%\right)=5.439\times 10^{-10}\times mV^{3}-2.731\times 10^{-6}\times mV^{2}+4.868\times 10^{-3}\times mV-2.683$$

**Figure 11:**
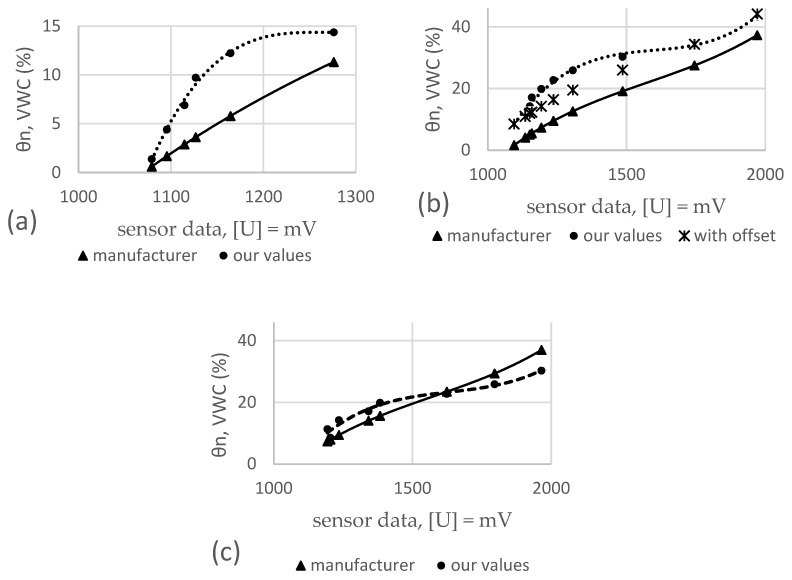
Measured and calculated VWC for TEROS 10 in substrates S1 (**a**), S2 (**b**), and S3 (**c**).

Figure 11a–c shows the calculated values for $\theta_{n}$, VWC (%) in comparison to each other for substrate S2. Both datasets again show a comparable slope and a relatively evenly offset that indicates a systematic error caused by our experiment design, the sensor environment, and installation/insertion. As in Figure 10b, we added an offset to the dataset calculated from the manufacturer’s equation and plotted it in Figure 11b. However, as can be seen, a significant deviation occurred approximately between 10 and 30% VWC, and even with an offset applied, the estimation of VWC would not be sufficient. Comparing the curves with the area of water available for plants shown in Figure 9, it can be seen that the manufacturer’s equation would lead to an underestimation of up to 20% VWC, which would lead to overwatering which would further lead to inefficiency and the washing out of nutrients and fine components of the substrate. When looking at the graphs for substrate S1 in Figure 11a, an even higher deviation between measured and calculated values can be seen. However, when looking at Figure 11c, a different picture emerges. Both curves look basically similar and would be almost identical if one of the two curves was rotated approximately around its center. The respective values and parameters are shown in Table 15. The calculated average deviations are shown in Table 16, with 3.9 ± 2.0 for S1, 9.8 ± 2.7 for S2, and 3.4 ± 1.9% VWC for S3.

### 4.3. DF Robot

**Figure 12:**
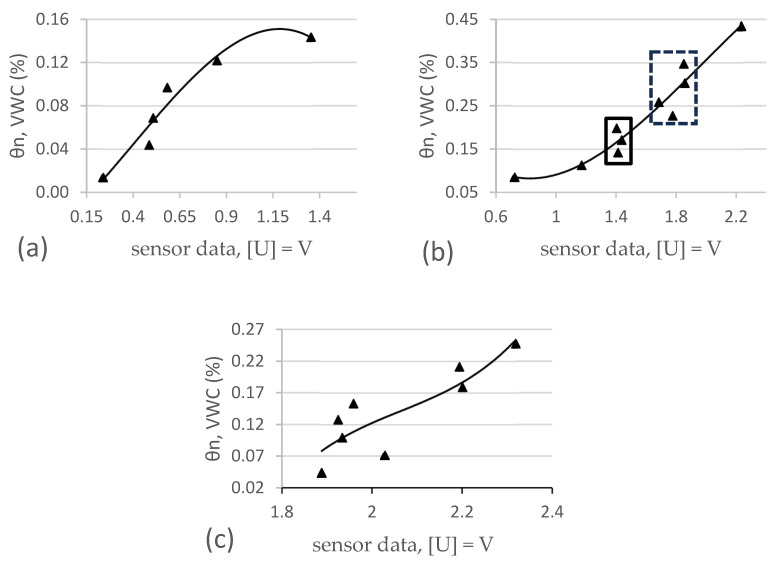
Measured and calculated VWC for DF Robot in substrates S1 (**a**), S2 (**b**), and S3 (**c**).

For DF Robot sensors, the manual only provides information about how the raw sensor data (digital values) can be interpreted as dry, wet, and very wet. Besides that, no information about calibration is given. Our results confirm this situation, as shown in Figure 12. Sensor values seem to be clustered, and the sensor is showing unpredictable behavior. However, when keeping in mind that the manufacturer states that the sensor can only be used to differentiate between dry, wet, and very wet, where dry is measured in air and very wet is measured in pure water, the sensor is essentially performing as expected. We have identified two data clusters in Figure 12b (bold square, dashed square). The data in the bold square extend over a VWC of approximately 23 to 29%, and the data within the dashed square extend over a VWC of approximately 32 to 44%. If we compare this to the suction stress curve of S2, it shows that 23 to 29% VWC lies in the range of water available to plants and that 32 to 44% VWC is beyond field capacity. This leads to the conclusion that a “wet” sensor reading effectively indicates that the plants have just the right amount of water and a “very wet” reading indicates that field capacity is reached; therefore, no watering should be carried out. This pattern can also be seen in substrate S3 (Figure 12c). In substrate S1, shown in Figure 12a, however, the sensor response can be well described by a cubic equation (see Table 5), since the clustering of values occurred to a lesser extent.

### 4.4. Scanntronik

**Figure 13:**
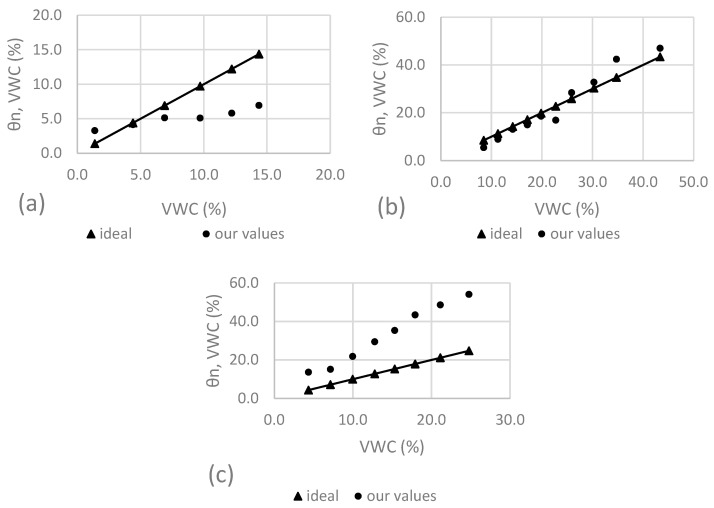
Measured and calculated VWC for Scanntronik in substrates S1 (**a**), S2 (**b**), and S3 (**c**).

Since the Scanntronik measurement system relies on proprietary data loggers that do not show any kind of raw data but directly show VWC values, and since no additional information is provided regarding calibration other than the fact that 0% VWC is assumed when the sensor is not in contact with water and 100% VWC is assumed when the sensor is submerged in pure water, we chose to directly look at the data shown in Figure 6, Figure 7 and Figure 8 but apply a linear fit to examine the deviations and compare these curves to ideal curves, where the real VWC, determined by weighing during our experiments, is equated with the VWC indicated by the sensor. As can be seen in Figure 13, only the values of the measurements in S1 (a) and S2 (b) seem to correlate with the real VWC (determined by weighing). This can also be seen from the calculated deviations and corresponding mean values, shown in Table 17, where the measurements show an average deviation of 3.7 ± 2.6 in S1, 3.2 ± 2.0 in S2, and 18.5 ± 7.9% VWC in S3.

## 5. Conclusions

Based on our results and analyses, the following questions regarding the sensors can be answered:

- Which sensor was least influenced by the insertion process and offered the most reproducible values between insertions?

The sensor least affected by the insertion process and providing the most reproducible values between insertions was TEROS 10. It exhibited the lowest average relative deviation and the smallest range of relative deviations across all tested substrates. This indicates that TEROS 10 delivers consistent measurements when the insertion technique remains the same. Interestingly, DFROBOT, the least expensive sensor in the study, showed the second lowest, though the differences between DFROBOT, SMT50, and Scanntronik in S3 were not significant. SMT50 and Scanntronik displayed similar values for the average of relative deviations with SMT50 performing slightly better in S2 and S3. However, the large range of relative deviations in most cases suggests that differences between sensors are not always statistically significant. For instance, in S1, comparing SMT50 and DFROBOT shows that no reliable conclusion can be drawn due to high variability. In S2, SMT50 exhibited a lower average of relative deviations than Scanntronik but with a higher standard deviation. Comparing SMT50 and DFROBOT, the performance of SMT50 could be as good as DFROBOT or vice versa. In S3, all sensors except TEROS 10 performed similarly, even when considering the variation in the average of relative deviation.

- Which sensor performs better on which substrate type and how does the same sensor perform on different substrates?

In principle, it is relatively difficult to compare substrates from different manufacturers, as the products are mixed from a wide variety of components and may belong to different categories. The substrates used in our tests can be categorized as mineral (S1) and organic substrate (S2, S3). The average deviation of the sensors, calculated from the deviations shown in Table 4, Table 6, and Table 8, respectively, are shown in Table 18. TEROS 10 showed the least variability between single measurements in all types of substrates. SMT 50 and Scanntronik showed the most variability of all sensors in S1 and S2. In S3, all sensors but TEROS 10 performed very similarly.

Table 19 shows the average deviations from the manufacturer’s equations (if available). As can be seen, all sensors have a relatively similar deviation in S1, TEROS 10 deviated the most in S2 (Scanntronik) and SMT 50 in S3.

- Have the values improved with the sieved substrate?

When looking at the averages shown in Table 18, as expected, the reduction in the average particle size through sieving seems to reduce the variation in sensor readings. This is probably because the substrate becomes more homogeneous, compact, and compressible and contact with the sensors is better guaranteed. In the case of TEROS 10, no significant improvement was achieved since the average deviations were already relatively low when measuring in the substrate that was not sieved (S2); moreover, the type of substrate had no significant influence on the average variation in general. In the case of SMT 50 and Scanntronik, the deviation was nearly halved, and an improvement is clearly visible.

- In practice, does the sensitivity difference in sensors matter for plants?

As mentioned above, we have evaluated the suitability of the sensors for use in irrigation systems using the suction pressure curve of S2, and the following conclusions are based on this comparison. In general, all examined sensors were able to cover the needed span of moisture where water is available for plants. To determine suitability, the deviations between the individual measurements at the same humidity are neglected, and only the deviations from the true values, which were determined gravimetrically, were considered. The behavior of the sensors can be divided into overestimation, where too high a moisture content is predicted and, therefore, not enough water is provided, and underestimation, where too low a moisture content is predicted and overwatering occurs. Overestimation can lead to the wilting or loss of plants and underestimation can lead to the loss of the nutrients and small particles of the substrate due to washing out. The predictions are either derived from equations provided by the manufacturer (SMT 50, TEROS 10) or directly from the sensor and datalogger unit (Scanntronik). In S2, only TEROS 10 showed significant underestimation (average of 9.8% VWC), and SMT 50 and Scanntronik showed only small underestimation (~3–4% VWC). In S1, SMT 50 and TEROS 10 showed significant underestimation, and Scanntronik showed overestimation. In S3, SMT 50 showed overestimation and Scanntronik showed underestimation.

## Figures and Tables

**Table 1:** Soil moisture sensors used in measurements.

Sensor Model	Manufacturer	Dimensions (L × W × H) [mm]	Measurement Method	Price [Euro] ^1^
TEROS 10	METER Group, Inc. Pullman, WA, USA	51 × 24 × 75	capacitive, FDR	160,-
Scanntronik	Scanntronik Mugrauer GmbH Zorneding, Germany	36 × 16 × 114	capacitive	189,-
Truebner, SMT 50	TRUEBNER GmbH Neustadt an der Weinstraße, Germany	21.5 × 16 × 95	capacitive, FDR	69,-
DF Robot	DFRobot Shanghai, China	30 × 15 × 175	capacitive	14,-

^1^ In the year 2024.

**Table 2:** Technical vegetation data of substrate in use.

	Mineral Content	Salinity (KCl)	pH-Value (CaCl_2_)	Density ^1^
	%	g/L	-	kg/m^3^
S1	100	ca. 0.1	ca. 7.5	1000–1150 ^2^
S2	30	<3.0	5.5–7.5	800–1000

^1^ density at maximum water saturation, ^2^ in a compacted state

**Table 3:** Installation depth of the sensors during calibration processes.

Sensor Insertion Depth	
Sensor	Insertion Depth ^1^ (cm)
TEROS 10	~8–16
SMT 50	~5–15
DFRobot	~0–18
Scanntronik	~6–17

^1^ measured from the top of the substrate.

**Table 4:** Recorded raw sensor data for substrate S1 determined during the experiment and calculated average Ø and standard deviation σ.

$S1,\;\mathrm{Recorded}\;\mathrm{Sensor}\;\mathrm{Values}\;\mathrm{for}\;\mathrm{Each}\;\mathrm{Moisture}\;\mathrm{Level}\;\mathrm{Examined}\;(\theta_{n})\;$*							
$\theta_{n}$ (%)		1.4	4.4	6.9	9.7	12.2	14.4
SMT50		0.1882	0.1997	0.2114	0.2233	0.2695	0.2695
		0.1301	0.2113	0.2229	0.2232	0.2463	0.3044
		0.1536	0.1765	0.2232	0.2461	0.2579	0.2811
		0.1652	0.1765	0.2232	0.2349	0.2579	0.3391
	Ø ^1^ ± σ	0.1593 ± 0.0209	0.1910 ± 0.0151	0.2202 ± 0.0051	0.2319 ± 0.0095	0.2579 ± 0.0082	0.2985 ± 0.0266
	σ (%)	13.15	7.89	2.30	4.09	3.18	8.91
DF Robot		2.7531	2.5647	2.5040	2.4081	2.2525	1.8676
		2.7513	2.5850	2.5057	2.4091	2.2521	1.6812
		2.7702	2.4191	2.4919	2.4400	2.1770	1.5981
		2.7710	2.4911	2.4737	2.4147	1.9211	1.4430
	Ø ^1^ ± σ	2.7614 ± 0.0092	2.5150 ± 0.0655	2.4938 ± 0.0128	2.4180 ± 0.0130	2.1507 ± 0.1361	1.6475 ± 0.1532
	σ (%)	0.33	2.60	0.51	0.54	6.33	9.30
TEROS 10		1.0684	1.0980	1.1021	1.1106	1.1522	1.2047
		1.0845	1.0885	1.1156	1.1253	1.1636	1.2743
		1.0746	1.1088	1.1267	1.1361	1.1685	1.2950
		1.0896	1.0872	1.1140	1.1355	1.1731	1.3302
	Ø ^1^ ± σ	1.0793 ± 0.0083	1.0956 ± 0.0087	1.1146 ± 0.0087	1.1269 ± 0.0103	1.1644 ± 0.0078	1.2761 ± 0.0458
	σ (%)	0.77	0.80	0.78	0.92	0.67	3.59
Scanntronik		3.4	4.0	4.8	4.9	5.3	7.3
		3.3	4.6	5.2	5.4	6.2	6.3
		3.3	3.3	5.3	4.8	5.9	7.6
		3.1	4.7	5.2	5.3	5.8	6.5
	Ø ^1^ ± σ	3.3 ± 0.1	4.2 ± 0.6	5.1 ± 0.2	5.1 ± 0.3	5.8 ± 0.3	6.9 ± 0.5
	σ (%)	3.33	13.47	3.75	4.50	5.59	7.80

* Values for SMT50, DF Robot, TEROS 10 in volts and Scanntronik in % VWC, ^1^ calculated from absolute values.

**Table 5:** The coefficients of the functions ^1^ of sensors in S1 and the coefficient of determination R^2^.

Sensor Model	a	b	c	d	R^2^
SMT 50	−73.508	48.641	−9.4275	0.5791	0.9916
DF Robot	−0.1085	0.1277	0.1547	−0.031	0.9491
TEROS 10	21.273	−80.576	101.73	−42.67	0.9933
Scanntronik	−0.0041	0.0608	−0.2518	0.3306	0.9595

^1^ y = ax^3^ + bx^2^ + cx + d.

**Table 6:** Values for substrate S2 were determined by the experiment and calculated average Ø and standard deviation σ.

$S2,\;\mathrm{Recorded}\;\mathrm{Sensor}\;\mathrm{Values}\;\mathrm{for}\;\mathrm{Each}\;\mathrm{Moisture}\;\mathrm{Level}\;\mathrm{Examined}\;(\theta_{n})\;$*											
$\theta_{n}$ (%)		8.5	11.3	14.2	17.1	19.9	22.7	25.9	30.3	34.8	43.4
SMT 50		0.2579	0.4437	0.4901	0.9196	1.0125	1.1750	1.0937	1.4652	2.9743	2.2430
		0.2463	0.4320	0.5836	0.6062	1.0125	1.1401	1.4071	1.4071	1.7322	2.9743
		0.2463	0.4785	0.5365	0.9196	0.9080	1.4304	1.4652	1.4652	2.3823	1.9179
		0.2579	0.5017	0.5249	0.7570	-	1.5929	-	2.1153	1.5696	1.8482
	Ø ^1^ ± σ	0.2521 ± 0.0058	0.4640 ± 0.0277	0.5338 ± 0.0335	0.8006 ± 0.1304	0.9777 ± 0.0493	1.3346 ± 0.1865	1.3220 ± 0.1632	1.6132 ± 0.2909	2.1646 ± 0.5577	2.2459 ± 0.4462
	σ (%)	2.30	5.97	6.27	16.29	5.04	13.98	12.34	18.03	25.76	19.87
DF Robot		2.2350	1.7736	1.693	1.4546	1.6290	0.9821	1.6437	1.0334	1.2112	0.7450
		2.3428	1.8103	1.4989	1.6865	1.7087	1.2712	1.2712	1.332	1.445	0.8206
		2.2782	1.9247	1.6141	1.5397	1.5287	1.3135	1.2662	1.1503	0.9400	0.8255
		2.2490	1.8141	1.5456	1.5765	1.5217	1.3295	1.0864	1.0600	1.0056	0.6790
	Ø ^1^ ± σ	2.2763 ± 0.0415	1.8307 ± 0.0565	1.5879 ± 0.0732	1.5643 ± 0.0832	1.5970 ± 0.0772	1.2241 ± 0.1413	1.3169 ± 0.2028	1.1439 ± 0.1169	1.1505 ± 0.1973	0.7675 ± 0.0603
	σ (%)	1.82	3.09	4.61	5.32	4.83	11.54	15.40	10.22	17.15	7.85
TEROS 10		1.0848	1.1360	1.1383	1.1504	1.2132	1.2155	1.3072	1.4455	1.6628	2.0066
		1.0952	1.1199	1.1572	1.1674	1.1932	1.2211	1.3200	1.4562	1.7846	2.0568
		1.0985	1.1415	1.1431	1.1646	1.1726	1.2618	1.2915	1.5385	1.7878	1.8499
		1.1002	1.1406	1.1673	1.1513	-	1.2481	-	1.5039	-	-
	Ø ^1^ ± σ	1.0947 ± 0.0060	1.1345 ± 0.0087	1.1515 ± 0.0115	1.1584 ± 0.0076	1.1930 ± 0.0166	1.2366 ± 0.0191	1.3062 ± 0.0117	1.4860 ± 0.0374	1.7451 ± 0.0582	1.9711 ± 0.0881
	σ (%)	0.55	0.77	1.00	0.66	1.39	1.54	0.89	2.52	3.33	4.47
Scanntronik		4.2	8.0	13.3	15.9	23.6	18.2	28.2	21.9	37.5	49.5
		5.4	9.1	13.5	17.1	13.9	12.6	25.6	38.2	45.2	36.4
		5.1	9.5	10.4	13.1	15.5	15.7	27.7	33.1	39.8	56.9
		7.1	9.2	15.3	13.8	21.4	21.1	32.3	38.0	47.2	45.2
	Ø ^1^ ± σ	5.5 ± 1.1	9.0 ± 0.6	13.1 ± 1.8	15.0 ± 1.6	18.6 ± 4.0	16.9 ± 3.1	28.5 ± 2.4	32.8 ± 6.6	42.4 ± 3.9	47.0 ± 7.4
	σ (%)	19.3	6.3	13.4	10.7	21.6	18.5	8.5	20.2	9.3	15.8

* Values for SMT50, DF Robot, TEROS 10 in V and Scanntronik in % VWC, ^1^ calculated from absolute values.

**Table 7:** The coefficients of the functions ^1^ of sensors in S2 and the coefficient of determination R^2^.

Sensor Model	a	b	c	d	R^2^
SMT 50	0.0273	−0.0944	0.2419	0.0272	0.9690
DF Robot	−0.0861	0.5128	−0.6702	0.3343	0.9406
TEROS 10	1.576	−7.5523	12.143	−6.2261	0.9916
Scanntronik	0.000006	−0.0005	0.0192	−0.0152	0.9765

^1^ y = ax^3^ + bx^2^ + cx + d.

**Table 8:** Values for substrate S3 determined by the experiment and calculated average Ø and standard deviation σ.

$S2,\;\mathrm{Recorded}\;\mathrm{Sensor}\;\mathrm{Values}\;\mathrm{for}\;\mathrm{Each}\;\mathrm{Moisture}\;\mathrm{Level}\;\mathrm{Examined}\;(\theta_{n})\;$*									
$\theta_{n}$ (%)		4.4	7.1	10.0	12.8	15.3	17.9	21.1	24.8
SMT 50		0.5945	0.8383	1.0474	1.7324	1.8484	2.3823	2.9742	-
		0.5713	0.8499	1.1980	1.7904	2.3126	2.9743	2.9742	-
		0.8035	0.7570	1.0472	2.1151	2.1734	2.9742	2.9742	-
		0.5829	0.7222	1.0474	1.8481	2.3125	2.9742	2.9742	-
	Ø ^1^ ± σ	0.6381 ± 0.0959	0.7919 ± 0.0538	1.0850 ± 0.0652	1.8715 ± 0.1465	2.1617 ± 0.1896	2.8263 ± 0.2563	2.9742 ± 0	-
	σ (%)	15.03	6.80	6.01	7.83	8.77	9.07	0.00	-
DF Robot		1.1583	1.0840	1.0025	1.1290	1.0560	0.8640	0.8760	0.6934
		1.0875	0.7787	1.0821	1.1302	1.1637	0.7606	0.7644	0.7061
		1.1248	0.9544	1.1833	0.9628	0.8392	0.8224	0.8413	0.7036
		1.0771	1.0701	0.9985	1.081	1.1073	0.7519	0.7446	0.6224
	Ø ^1^ ± σ	1.1119 ± 0.0321	0.9718 ± 0.1223	1.0666 ± 0.0752	1.0758 ± 0.0682	1.0416 ± 0.1229	0.7997 ± 0.0460	0.8066 ± 0.0540	0.6814 ± 0.0344
	σ (%)	2.89	12.59	7.05	6.34	11.80	5.75	6.69	5.05
TEROS 10		1.1973	1.2123	1.2443	1.3008	1.3828	1.6339	1.7931	1.8971
		1.2077	1.1874	1.2443	1.3360	1.3816	1.5847	1.8509	1.9871
		1.2038	1.1919	1.2306	1.3393	1.3828	1.6537	1.7364	1.9993
		1.2073	1.1809	1.2189	1.3892	1.3840	1.6227	1.8033	1.9762
	Ø ^1^ ± σ	1.2040 ± 0.0042	1.1931 ± 0.0117	1.2345 ± 0.0106	1.3413 ± 0.0315	1.3828 ± 0.0008	1.6238 ± 0.0251	1.7959 ± 0.0407	1.9649 ± 0.0400
	σ (%)	0.35	0.98	0.86	2.35	0.06	1.55	2.27	2.04
Scanntronik		11.9	15.3	21.5	27.4	25.8	44.4	47.2	58.0
		14.4	14.9	20.7	32.6	32.9	40.9	46.2	48.8
		13.6	15.7	20.9	25.6	40.8	42.1	49.0	50.8
		14.5	14.5	24.1	32.1	41.9	46.4	52.0	58.8
	Ø ^1^ ± σ	13.6 ± 1.0	15.1 ± 0.4	21.8 ± 1.4	29.4 ± 3.0	35.4 ± 6.5	43.5 ± 2.1	48.6 ± 2.2	54.1 ± 4.4
	σ (%)	7.7	3.0	6.2	10.2	18.4	4.9	4.5	8.1

* Values for SMT50, DF Robot, TEROS 10 in V and Scanntronik in % VWC, ^1^ calculated from absolute values.

**Table 9:** The coefficients of the functions ^1^ of sensors in S3 and the coefficient of determination R^2^.

Sensor Model	a	b	c	d	R^2^
SMT 50	0.0436	−0.2359	0.4424	−0.1546	0.9694
DF Robot	2.3456	−14.524	30.263	−21.073	0.7452
TEROS 10	0.9415	−4.64	7.7163	−4.1403	0.9693
Scanntronik	0.000004	−0.0004	0.0159	−0.1046	0.9948

^1^ y = ax^3^ + bx^2^ + cx + d.

**Table 10:** Average relative deviation of raw sensor values in S1.

$\mathrm{Average}\;\mathrm{Relative}\;\mathrm{Deviation}\;x_{avg}$ of Raw Sensor Values in S1		
Sensor Model	$x_{\min}-x_{max}$	$x_{avg}\pm \sigma$
SMT50	2.30–13.15	6.59 ± 3.79
DFROBOT	0.33–9.30	3.27 ± 3.41
TEROS 10	0.67–3.59	1.26 ± 1.05
Scanntronik	3.33–13.47	6.41 ± 3.48

**Table 11:** Average relative deviation of raw sensor values in S2.

$\mathrm{Average}\;\mathrm{Relative}\;\mathrm{Deviation}\;x_{avg}$ of Raw Sensor Values in S2		
Sensor Model	$x_{\min}{-x}_{max}$	$x_{avg}\pm \sigma$
SMT50	2.30–25.76	12.59 ± 7.20
DFROBOT	1.82–17.15	8.18 ± 4.97
TEROS 10	0.55–4.47	1.71 ± 1.25
Scanntronik	6.30–21.60	14.36 ± 5.19

**Table 12:** Average relative deviation of raw sensor values in S3.

$\mathrm{Average}\;\mathrm{Relative}\;\mathrm{Deviation}\;x_{avg}$ of Raw Sensor Values in S3		
Sensor Model	$x_{\min}{-x}_{max}$	$x_{avg}\pm \sigma$
SMT50	0.00–15.03	7.64 ± 4.14
DFROBOT	2.89–12.59	7.27 ± 3.09
TEROS 10	0.06–2.35	1.31 ± 0.82
Scanntronik	3.00–18.40	7.88 ± 4.51

**Table 13:** Comparison of the coefficients of the functions ^1^ in Figure 10 and the coefficient of determination R^2^.

	Manufacturer			Our Values		
	S1	S2	S3	S1	S2	S3
a	16.667	16.667	16.667	98.232	15.579	7.278
b	1 × 10^−14^	3 × 10^−14^	3 × 10^−14^	−14.087	4.5584	4.7709
R^2^	1	1	1	0.9726	0.9659	0.9309

^1^ y = ax + b.

**Table 14:** Comparison of average Ø and difference Δ between calculated and experimental VWC for SMT 50.

$\theta_{n}$ , VWC (%)									
	S1			S2			S3		
	Manufacturer	Our Values	Δ	Manufacturer	Our Values	Δ	Manufacturer	Our Values	Δ
	1.4	2.7	1.3	8.5	4.2	−4.3	8.5	10.6	2.1
	4.4	3.2	−1.2	11.3	7.7	−3.6	11.3	13.2	1.9
	6.9	3.7	−3.2	14.2	8.9	−5.3	14.2	18.1	3.9
	9.7	3.9	−5.8	17.1	13.3	−3.8	17.1	31.2	14.1
	12.2	4.3	−7.9	19.8	16.3	−3.6	19.8	36.0	16.2
	14.4	5.0	−9.4	22.7	22.2	−0.5	22.7	47.1	24.4
	-	-	-	25.9	22.0	−3.8	25.9	49.6	23.7
	-	-	-	30.3	26.9	−3.4	30.3	50.0	19.7
	-	-	-	34.7	36.1	1.3	-	-	-
	-	-	-	43.4	37.4	−5.9	-	-	-
Ø ^1^	-	-	4.8 ± 3.2	-	-	3.6 ± 1.5	-	-	13.2 ± 8.8

^1^ Calculated from absolute values.

**Table 15:** Comparison of the coefficients of the functions ^1^ in Figure 11 and the coefficient of determination R^2^.

	Manufacturer	Our Values		
		S1	S2	S3
a	5 × 10^−8^	2 × 10^−6^	2 × 10^−7^	1 × 10^−7^
b	0.0002	−0.0081	−0.0008	−0.0005
c	0.3898	10.173	1.2143	0.8287
d	−215.4	−4267	−622.61	−439.12
R^2^	1	0.9933	0.9916	0.974

^1^ y = ax^3^ + bx^2^ + cx + d.

**Table 16:** Comparison of average Ø and difference Δ between calculated and experimental VWC for TEROS 10.

$\theta_{n}$, VWC (%)									
	S1			S2			S3		
	Manufacturer	Our Values	Δ	Manufacturer	Our Values	Δ	Manufacturer	Our Values	Δ
	1.4	0.6	−0.8	8.5	1.6	−6.9	8.5	7.9	−0.6
	4.4	1.7	−2.7	11.3	4.1	−7.2	11.3	7.3	−4.0
	6.9	2.9	−4.0	14.2	5.1	−9.2	14.2	9.4	−4.8
	9.7	3.6	−6.1	17.1	5.4	−11.7	17.1	14.0	−3.1
	12.2	5.8	−6.4	19.8	7.3	−12.5	19.8	15.6	−4.3
	14.4	11.3	−3.1	22.7	9.5	−13.2	22.7	23.5	0.7
	-	-	-	25.9	12.6	−13.3	25.9	29.3	3.5
	-	-	-	30.3	19.1	−11.2	30.3	37.0	6.7
	-	-	-	34.7	27.5	−7.3	-	-	-
	-	-	-	43.4	37.3	−6.1	-	-	-
Ø ^1^	-	-	3.9 ± 2.0	-	-	9.8 ± 2.7	-	-	3.4 ± 1.9

^1^ Calculated from absolute values.

**Table 17:** Comparison of average Ø and difference Δ between calculated and experimental VWC for Scanntronik.

$\theta_{n}$ , VWC (%)									
	S1			S2			S3		
	Ideal	Sensor Values	Δ	Ideal	Sensor Values	Δ	Ideal	Sensor Values	Δ
	1.4	3.3	1.9	8.5	5.5	−3.0	4.3	13.6	9.3
	4.4	4.2	−0.2	11.3	9.0	−2.3	7.1	15.1	8.0
	6.9	5.1	−1.8	14.2	13.1	−1.1	10.0	21.8	11.8
	9.7	5.1	−4.6	17.1	15.0	−2.1	12.8	29.4	16.6
	12.2	5.8	−6.4	19.8	18.6	−1.2	15.3	35.4	20.0
	14.4	6.9	−7.4	22.7	16.9	−5.8	17.9	43.5	25.5
	-	-	-	25.9	28.5	2.6	21.1	48.6	27.5
	-	-	-	30.3	32.8	2.5	24.8	54.1	29.3
	-	-	-	34.7	42.4	7.7	-	-	-
	-	-	-	43.4	47.0	3.6	-	-	-
Ø ^1^	-	-	3.7 ± 2.6	-	-	3.2 ± 2.0	-	-	18.5 ± 7.9

^1^ Calculated from absolute values.

**Table 18:** Calculated average of the average deviation between individual measurements for each sensor and substrate combination.

Substrate	S1	S2	S3
SMT 50	6.59 ± 3.79	12.58 ± 7.20	7.64 ± 4.13
DF Robot	3.27 ± 3.41	8.18 ± 4.97	7.27 ± 3.09
TEROS 10	1.25 ± 1.05	1.71 ± 1.25	1.31 ± 0.82
Scanntronik	6.49 ± 3.44	14.36 ± 5.18	7.87 ± 4.53

All values in % VWC; values for calcualtion taken from Table 4, Table 6 and Table 8.

**Table 19:** Calculated average of the deviation between calculated VWC from manufacturer’s equation and experimental values for each sensor and substrate combination.

Substrate	S1	S2	S3
SMT 50	4.8 ± 3.2	3.6 ± 1.5	13.2 ± 8.8
DF Robot	-	-	-
TEROS 10	3.9 ± 2.0	9.8 ± 2.7	3.4 ± 1.9
Scanntronik	3.7 ± 2.6	3.2 ± 2.0	18.5 ± 7.9

All values in % VWC; values for calculation taken from Table 15, Table 16 and Table 17.

## Data Availability

The data presented in this study is available on request from the corresponding author.
